# The Biggest Loser Thinks Long-Term: Recency as a Predictor of Success in Weight Management

**DOI:** 10.3389/fpsyg.2015.01864

**Published:** 2015-12-08

**Authors:** Gilly Koritzky, Chantelle Rice, Camille Dieterle, Antoine Bechara

**Affiliations:** ^1^American School of Professional Psychology, Argosy University, Orange CountyOrange, CA, USA; ^2^Division of Occupational Science and Occupational Therapy, University of Southern CaliforniaLos Angeles, CA, USA; ^3^Department of Psychology, University of Southern California, Los AngelesCA, USA; ^4^Department of Neurology, University of IowaIowa City, IA, USA

**Keywords:** Iowa Gambling Task, obesity, weight management, recency, weight loss, decision making, long term thinking, Expectancy-Valence model

## Abstract

Only a minority of participants in behavioral weight management lose weight significantly. The ability to predict who is likely to benefit from weight management can improve the efficiency of obesity treatment. Identifying predictors of weight loss can also reveal potential ways to improve existing treatments. We propose a neuro-psychological model that is focused on recency: the reliance on recent information at the expense of time-distant information. Forty-four weight-management patients completed a decision-making task and their recency level was estimated by a mathematical model. Impulsivity and risk-taking were also measured for comparison. Weight loss was measured in the end of the 16-week intervention. Consistent with our hypothesis, successful dieters (*n* = 12) had lower recency scores than unsuccessful ones (*n* = 32; *p* = 0.006). Successful and unsuccessful dieters were similar in their demographics, intelligence, risk taking, impulsivity, and delay of gratification. We conclude that dieters who process time-distant information in their decision making are more likely to lose weight than those who are high in recency. We argue that having low recency facilitates future-oriented thinking, and thereby contributes to behavior change treatment adherence. Our findings underline the importance of choosing the right treatment for every individual, and outline a way to improve weight-management processes for more patients.

## Introduction

Obesity and its adverse effects on health are becoming increasingly prevalent in the United States as well as worldwide (World Health Organization, 2015). Behavioral interventions in weight management, i.e., programs that target energy balance-related behaviors (eating habits and physical activity) to promote weight loss, are very limited in their success. An extensive meta-analysis concludes that the mean weight loss following 6 months of intervention only ranges from 5 to 9% (Franz et al., 2007). While health professionals agree that even a modest loss of 5–10% of one's weight is beneficial (e.g., Diabetes Prevention Program Research Group, 2004; Centers for Disease Control and Prevention, 2012; Look AHEAD Research Group, 2014), most accounts of weight-loss programs' effectiveness show that the majority of participants do not even achieve this goal (e.g., Heshka et al., 2003; Appel et al., 2011). The few interventions that have higher success records typically include intensive lifestyle change and/or meal replacement (e.g., Heymsfield et al., 2003; Diabetes Prevention Program Research Group, 2004; Befort et al., 2010; Look AHEAD Research Group, 2014). In other words, higher costs are involved in achieving stronger effects. More importantly, it is evident that no intervention results in significant weight loss for all participants (Wing, 2003).

The ability to predict who is likely to benefit from weight management can greatly improve the efficiency of obesity treatment (Teixeira et al., 2005; Schwartz and Brownell, 1995; Expert Panel on the Identification, Treatment of Overweight, and Obesity in Adults (US), 2002). It will enable patients and health professionals to make informed choices between available treatment types, and to recommend behavioral intervention to those most likely to benefit from it (for example, instead of or before turning to bariatric surgery). This will help save time and resources and reduce patients' distress. Moreover, identifying predictors of successful weight management can shed light on what hinders it for some patients, and potentially lead to developing solutions that will fit their needs as well.

Many studies of weight management outcomes report some correlates of successful weight loss. Yet these correlates are often routinely recorded variables such as gender (e.g., Kiernan et al., 1998), initial weight (e.g., Traverso et al., 2000), or previous dieting (e.g., Teixeira et al., 2002). Psychological constructs, which suggest potential explanations of weight-loss success, have been studied as well. Among these are emotional eating (Bryan and Tiggemann, 2001) or eating in response to external cues (Traverso et al., 2000), eating-related cognitive restraint (Foster et al., 1998), perceived hunger (Teixeira et al., 2002), body image (Traverso et al., 2000), self-esteem (Bryan and Tiggemann, 2001), locus of control (Williams et al., 1996), social support (Kiernan et al., 1998), and self-motivation or general efficacy (Williams et al., 1996; Teixeira et al., 2002; Palmeira et al., 2007). However, a comprehensive review concludes that the evidence is mixed with respect to most of these constructs except for the latter (Teixeira et al., 2005).

Contemporary neuropsychological theories argue that obesity involves dysfunctional dynamics between reward-seeking drives, on one hand, and failing inhibitory control, on the other (Epstein et al., 2010; Carr et al., 2011). These theories hold that excessive consumption of food tends to be associated with a decision-making setback, in which immediate gratification supersedes long-term considerations regarding health risks. In light of findings from brain studies, we previously argued (Bechara and Damasio, 2005; Hochman et al., 2010; Koritzky et al., 2013) that (A) the processing of immediate, certain, or tangible outcomes in the prefrontal cortex is triggered directly by brain structures that represent reward-driven motivation and affect, while (B) the processing of information about delayed, uncertain or intangible outcomes involves indirect and polysynaptic neural connections. This difference implies that the processing of time-distant outcomes (such as disease diagnosis) is more effortful than the processing of immediate outcomes (such as the pleasure of eating), which might lead to a tendency to make decisions based mostly on immediate considerations. This is manifested in poor inhibitory control, which is often found to be higher in obese individuals than in their normal-weight counterparts (Nederkoorn et al., 2006; Davis et al., 2007; Batterink et al., 2010; Koritzky et al., 2012). These findings suggest that obesity is generally associated with a tendency to base decisions on immediate considerations, but they do not necessarily imply a connection between this tendency and the odds of weight-loss success within the obese population. It seems plausible that reliance on immediate considerations will hinder weight-loss endeavors, and if it does, it can be a useful predictor of weight-management success.

However, unlike differences between obese patients and lean controls, cognitive differences within the obese population are more subtle and harder to detect. For example, common measures of impulsivity, delay discounting, cognitive function, or decision making impairments can differentiate between obese and non-obese subjects (Nederkoorn et al., 2006; Davis et al., 2007; Weller et al., 2008; Smith et al., 2011; Koritzky et al., 2012), but because obese individuals tend to obtain similar scores in them, these measures are often not sensitive to individual differences within the obese population (Bryan and Tiggemann, 2001; Koritzky et al., 2014). It follows that a novel measure is required in order to predict success in weight-management interventions that target this population.

In the present study we chose to estimate reliance on immediate considerations by the Expectancy-Valence model (EV; Busemeyer and Stout, 2002), a quantitative model that analyzes behavior in complex decision-making tasks. The EV Model is designed to capture individual differences in decision making and is known to differentiate well between subpopulations with decision-making deficits that are otherwise indistinguishable from one another (Yechiam et al., 2005, 2008). Moreover, past research has linked the EV Model's recency component (see below) to activation in the anterior prefrontal cortex, a region associated with effortful information processing and inhibitory control (Hochman et al., 2010; Koritzky et al., 2013).

The EV model specifies three underlying components of decision making: (1) a motivational component indicating the subjective weight the decision-maker assigns to gains versus losses; (2) a recency component indicating the extent to which one's decisions are affected by new information at the expense of taking all potential outcomes into account, and; (3) a probabilistic component indicating consistency. Based on an analysis of choice behavior during a decision-making task (typically the Iowa Gambling Task; Bechara et al., 1994), the model estimates three individual parameters corresponding to these components for each participant (Busemeyer and Stout, 2002).

We hypothesize that compared to unsuccessful dieters, successful dieters will show greater tendency to take time-distant (or long term) information into account in the process of decision making. That is, successful dieters will have lower scores in the recency parameter of the EV Model.

It might seem difficult to make a clear distinction between recency, or the processing of time-distant outcomes while making decisions, and such constructs as impulsivity or delay discounting. While these constructs are related to one another, they do not necessarily represent the same neuro-cognitive processes. For example, it has been argued that recency refers to information processing at an early stage of making a decision, while delay discounting reflects a preference that comes into play in a later stage (Busemeyer et al., in press). Similarly, there is mixed evidence with respect to the overlap between delay discounting and impulse control (e.g., Kirby et al., 1999; Reynolds et al., 2006). Although, this theoretical debate is important in its own right, it is not critical to the present study. That is, even if one views recency as a proxy of impulsivity, its practical potential in clinical populations (Yechiam et al., 2005, 2008) makes recency a predictor worth considering.

Indeed, past research has linked obesity with impulsivity (e.g., Davis et al., 2007; Batterink et al., 2010), delay of gratification (Weller et al., 2008), and elevated risk taking in decision-making (Koritzky et al., 2012), and it may be suggested that these constructs predict obese patients' weight management outcomes. Comparing between all of these constructs as potential predictors can also contribute to our understanding of how similar they are. Therefore we included these as additional measures as well.

## Methods

### Participants

Participants were adults enrolled in a weight-management program serving the university faculty, staff, and students. Program clients were informed about the study upon signing up for the program, and study participation was voluntary. The initial sample included 70 individuals, who formed about 25% of the program's clients at the time of the study. Out of these, 26 (37%) dropped out before completing the program and were excluded from further analysis. This attrition rate is comparable with the literature; see (Honas et al., 2003; Teixeira et al., 2004; De Panfilis et al., 2008; Moroshko et al., 2011). Predictors of attrition have been discussed elsewhere (Koritzky et al., 2014).

The analytical sample of 44 participants was similar in its characteristics to the population of program completers (see Table 1). The program's general population has been described in Koritzky et al. (2014).

**Table 1:** **Characteristics of the study's sample compared to the general population of participants who completed the weight-management program**.

	**Study sample (*N* = 44)**	**Program population (*N* = 672)**
% Women	81%	78%
% White	59%	51%
Age	45.6 (12.07)	46.0 (12.9)
% Lost weight successfully	27%	23%

The study was carried out in accordance with the guidelines set by the Institutional Review Board at the University of Southern California. All participants gave written informed consent in accordance with the Declaration of Helsinki.

### Procedure

Lifestyle Redesign® Weight-Management is an evidence-based program, which was developed by the Division of Occupational Science and Occupational Therapy at the University of Southern California. The program was 16 weeks long. Participants met weekly with an occupational therapist and received information about healthy diet and lifestyle, as well as personalized guidance. Height was measured in the beginning of the program, and weight was recorded weekly. No incentives were provided for weight-loss or other achievements.

Study participants attended a 1-h session in the beginning of the program, in which they completed the decision-making tasks and questionnaires described hereinafter. Participants were paid $20 on average for attending the session ($17 show-up fee and an additional amount of $1-$6 based on task performance; this is a standard procedure in decision-making studies whose purpose is to maintain participants' attention throughout the session (Kagel and Roth, 1995). Data about participants' weight change were obtained after the final meeting of the program. Because even a modest weight loss of 5% is likely to produce health benefits (Franz et al., 2007), and in accord with many other weight-loss protocols (e.g., Diabetes Prevention Program Research Group, 2004; Look AHEAD Research Group, 2014), we defined successful weight loss as losing at least 5% of one's initial weight.

### Main measures

#### The Iowa Gambling Task (Bechara et al., 1994)

A decision-making task designed to simulate real-life decisions in terms of conflict and complexity. Participants make repetitive choices between four decks of cards (displayed on a computer screen), with the goal of maximizing their earnings. Each card selection yields a gain, but occasionally losses occur too. Two of the decks are disadvantageous, in that they yield relatively high gains along with occasional losses that are even larger, resulting in a net loss. The two advantageous decks yield small gains combined with smaller losses, resulting in a net gain. High performance on the task depends on the subject's learning to prefer the advantageous decks, i.e., to select more from them than from the disadvantageous decks. The task had 100 trials. Task results were further analyzed using the Expectancy-Valence model (Busemeyer and Stout, 2002).

#### The expectancy-valence model (EV; Busemeyer and Stout, 2002)

According to the model, choices in complex environments are based on subjective expectancies, which reflect not only the actual outcomes experienced, but also individual differences in three components of the learning and decision process:

1. A motivational component indicating the subjective weight the decision-maker assigns to gains versus losses. The *sensitivity to reward* parameter ranges between 0 and 1, and represents the relative weight assigned to gains (rewards) in the evaluation of alternatives.
2. A learning-rate component indicating the degree of prominence given to recent outcomes at the expense of relying on the full range of past experience. The *Recency* parameter ranges between 0 and 1, and represents (inversely) the tendency to take long-term considerations into account (Koritzky et al., 2013).
3. A probabilistic component indicating how consistent the decision-maker is between learning and responding. The *Consistency* parameter ranges between 0 and 10 and represents the tendency to choose the alternatives with the higher subjective expectancies, as opposed to making random selections.

Based on a trial-to-trial analysis of behavior in the decision task, the model extracts three individual parameters corresponding to these components, for each decision maker.

### Additional measures

#### Simplified variant of the Iowa Gambling Task (Koritzky et al., 2012)

This version focuses on risk-taking tendencies. The advantageous decks produce a constant small gain, i.e., no risk. The disadvantageous decks produce either gains or losses, i.e., they entail considerable risk.

#### Barratt impulsiveness scale (Patton et al., 1995)

A self-report, 30-item questionnaire measuring impulsivity.

#### Food-specific Go/No Go task (Batterink et al., 2010)

A behavioral measure of impulsivity. In this task, a rapid stream of desserts' pictures or vegetables' pictures is displayed, and the participant needs to react as quickly and accurately as possible by pressing a key in response to vegetables, but not desserts. The task measures the ability to withhold, or inhibit, dominant behavior.

#### A delay of gratification task (see Newman et al., 1992)

In this task, participants repeatedly choose between two unmarked buttons displayed on a computer monitor. Buttons yield a small payoff of 5 points in either 40% (low frequency) or 80% (high frequency) of the trials. The low-frequency button is available for pressing as soon as each trial begins, while the high-frequency button becomes available after a 10-s delay. In each trial the participant chooses whether to wait the 10 s for better prospects of reward, or press the low-frequency button immediately and move to the next trial faster.

The Raven Advanced Progressive Matrices Test, part 1. A brief measure of intelligence.

#### Demographic questionnaire

Included items referring to gender, age, education, employment status, race and ethnicity, and dieting history.

### Statistical analysis

Comparisons between successful and unsuccessful dieters were done using *t*-test or fisher's exact test, as appropriate for each variable. Weight-loss success was predicted using logistic regression models with recency as the predictor. Weight loss was coded “1” for dieters who lost at least 5% of their initial body weight, and “0” otherwise. Because successful and unsuccessful dieters differed significantly in their reported number of past weight-loss attempts (see Table 2), we included this variable in an additional regression model. All *p*-values are two sided unless noted otherwise. Analyses were carried out using SAS 9.4 software.

**Table 2:** **Characteristics (means and S.D.) of successful and unsuccessful dieters**.

	**Successful (*n* = 12)**	**Unsuccessful (*n* = 32)**	
% Women	75%	84%	n.s.
% White	58%	45%	n.s.
Weight [lbs]	185.5 (36.95)	204.4 (51.43)	n.s.
Body Mass Index	30.95 (4.01)	33.82 (6.42)	n.s.
Age	46.42 (15.40)	45.28 (10.84)	n.s.
No. of weekly working hours	36.04 (9.03)	40.03 (11.11)	n.s.
Education level [% of participants with college degree]	92%	84%	n.s.
No. of prior weight-loss attempts	4.42 (3.15)	10.6 (9.97)	*p* < 0.01

## Results

### Participant characteristics

Study participants attended 15.57 (S.D. = 0.84) weekly meetings on average. The last recorded weight was used to calculate weight-loss percentage for each participant. Twelve participants (27%) lost 5% or more of their original weight, which satisfied the aforementioned criterion for successful weight loss, while 32 participants (73%) were unsuccessful (they either lost less than 5% of their weight, or their weight increased)^1^. This success rate is similar to others reported in the literature (e.g., Heshka et al., 2003; Appel et al., 2011). Table 2 provides the initial weight, BMI, and demographic characteristics of successful and unsuccessful participants. As can be seen, the differences between the groups were insignificant except for one variable. While all participants had tried to lose weight prior in the past, unsuccessful dieters reported a larger number of attempts [*t*_(38.9)_ = 3.04, *p* = 0.005].

### Main outcomes

On average, both groups performed the Iowa Gambling Task at a similar level. There was no difference in the number of advantageous choices made by successful (mean = 61%, S.D. = 18%) and unsuccessful (mean = 63%, S.D. = 20%) dieters.

Table 3 presents the EV model fit estimates and mean parameter scores. As hypothesized, recency scores were significantly lower in those who lost weight successfully than in unsuccessful dieters [*t*_(35.6)_ = −2.95, *p* = 0.006; Cohen's *d* = 0.89, indicating a large effect size]. A power analysis conducted with G^*^Power software revealed a power estimate of 0.826 for this result. The other two EV model parameters—consistency and sensitivity to reward—did not differ between the groups.

**Table 3:** **Means (S.D.) of the Expectancy-Valence model fit estimates and parameters in successful and unsuccessful dieters**.

	**Successful (*n* = 12)**	**Unsuccessful (*n* = 32)**	
Model fit	10.18 (17.66)	18.77 (32.34)	n.s.
Sensitivity to reward	0.56 (0.25)	0.55 (0.36)	n.s.
Recency	0.11 (0.24)	0.42 (0.43)	*p* < 0.01
Consistency	4.01 (4.01)	2.82 (2.76)	n.s.

The regression model for predicting weight-loss success based on recency was significant [Likelihood Ratio $\chi_{\left(1\right)}^{2}=5.96$, *p* = 0.015; Max-rescaled R-Square = 0.184]. The regression coefficient of the predictor was significant as well [$\chi_{\left(1\right)}^{2}=3.66$, *p* = 0.03, one sided]. These results indicate that success in a behavioral weight-management intervention is predicted (negatively) by the tendency to base decisions on immediate considerations.

Because the number of past dieting attempts was different between successful and unsuccessful dieters, we included it in a second regression model along with recency. This regression model had improved fit [Likelihood Ratio $\chi_{\left(1\right)}^{2}$ = 9.32, *p* = 0.001; Max-rescaled R-Square = 0.285], yet each coefficient only achieved marginal significance [recency: $\chi_{\left(1\right)}^{2}$ = 2.64, *p* = 0.052, one sided; number of past diets: $\chi_{\left(1\right)}^{2}$ = 2.27, *p* = 0.066, one sided].

### Additional outcomes

Risk-taking, impulsivity, delay of gratification, or intelligence did not predict weight-loss success in this sample. We found no significant differences between successful and unsuccessful dieters in the simplified variant of the Iowa Gambling Task [*t*_(42)_ = 1.28, *p* = 0.21], the Barratt Impulsiveness Scale [*t*_(42)_ = 0.06, *p* = 0.95], the delay of gratification task [*t*_(42)_ = 1.12, *p* = 0.27], the Go/No Go Task [*t*_(42)_ = 1.54, *p* = 0.13 for false alarms; *t*_(42)_ = 0.99, *p* = 0.33 for the sensitivity index d'; *t*_(42)_ = 0.23, *p* = 0.82 for the criterion], or the Raven Advanced Progressive Matrices Test [*t*_(42)_ = 0.49, *p* = 0.63].

## Discussion

Consistent with our hypothesis, weight loss in a weight management intervention is predicted by recency, or the rate of updating recent information in the process of decision making. Our findings support the notion that successful dieters tend to take time-distant (or long-term) information into account in their decision making, while unsuccessful dieters tend to rely more heavily on recent outcomes as a source of information. Moreover, recency was the only study variable that distinguished well between successful and unsuccessful dieters. Successful and unsuccessful dieters were similar in their demographics as well as psychometric characteristics such as intelligence, general decision-making performance, risk taking, impulsivity, and delay of gratification. The scarcity of valid predictors of weight management outcomes has been noted by others (Teixeira et al., 2005).

The present study presents a theoretically-grounded explanation for individual differences in weight-loss success. We argue that the ability to “think long term,” i.e., to think about the potential time-distant outcomes of one's actions, contributes significantly to behavioral change in the context of weight management. Patients in obesity treatment are attempting to acquire eating habits that reflect health concerns rather than gustatory satisfaction. Because satisfaction is achieved immediately while the risks associated with unhealthy eating are a probabilistic future consequence, the former is easier to think of or process than the latter (Bechara, 2005; Noël et al., 2013). Therefore, dieters who are better able to engage in this more difficult and effortful information processing are more likely to change their habits successfully and lose weight as a result.

At the neuropsychological level, individual differences in recency correspond to differences in the activation of the prefrontal cortex (Koritzky et al., 2013), a brain area that is linked with inhibitory control (e.g., Bechara, 2005). Interestingly, some studies have shown that obese patients who lost weight successfully display high activation in the prefrontal cortex when presented with food cues (McCaffery et al., 2009). This implies that they exert more inhibitory control, and potentially more intense long-term thinking (depending on the locus of the elevated activity within the prefrontal cortex; see Koritzky et al., 2013). These results are hard to interpret because they bring up the possibility that weight management causes improvement in long-term thinking rather than the other way around. However, our present findings suggest that high activation in the prefrontal cortex precedes weight-management success.

Notice that recency predicts weight loss among dieters who completed a weight management intervention, but it does not predict intervention completion: Attrition is captured by a different aspect of decision making: sensitivity to reward (Koritzky et al., 2014). Although, attrition from treatment and lack of success in it may represent difficulties in adherence to the treatment's requirements, these difficulties appear to bear on different cognitive processes, and, consequently, on different neural systems. In light of evidence that obesity resembles addiction (Volkow and Wise, 2005), we have argued (Koritzky et al., 2014) that obesity involves the same kind of dysfunctional dynamics between the brain-systems that are associated with decision making as addiction does (Bechara, 2005; Bickel et al., 2007; Goldstein and Volkow, 2011). The first such system is an *impulsive/motivational system* that promotes reward-driven behaviors. The second is a *reflective system* that modulates deliberation, forecasting of future consequences, and inhibitory control (Bechara, 2005; Bickel et al., 2007; Goldstein and Volkow, 2011). In this two-system model, the impulsive/motivational system is an abstraction of neural processes associated mainly with the amygdala and striatum, and the reflective system is an abstraction of neural processes associated mainly with the prefrontal cortex (Bechara, 2005). While the reflective system is associated with the *recency* parameter of the Expectancy-Valence model (Koritzky et al., 2013), the impulsive/motivational system has been associated with the sensitivity to reward parameter (Premkumar et al., 2008; Chan et al., 2014). Therefore these two components of the EV model—sensitivity to reward and recency—serve as behavioral measures of activation in two different neuropsychological systems.

It may be argued that our interpretation of recency as a marker of the ability to process potential future consequences is not very different from the concept of delay discounting. Indeed, these concepts seem hard to distinguish, but they are not identical. Recency refers to the rate of information updating, or learning, whereas delay discounting reflects a preference that comes into play in a different stage of decision making (Busemeyer et al., in press). In addition, in the present study weight loss was not predicted by delay of gratification, which is often interpreted as a measure of delay discounting (Kirby and Herrnstein, 1995).

Our results are consistent with previous studies that found having fewer previous weight-loss attempts predictive of weight-loss success (e.g., Teixeira et al., 2002). The direction of causality in the relationship between attempt-failure and number of past attempts is unclear, though. It has been suggested that a history of failed attempts reflects a physiological barrier to weight loss, which may be innate or developed through the years (Teixeira et al., 2005). In the terms of our theory, a neuro-cognitive property such as high recency may be such a barrier.

To date, the literature has not suggested many explanatory variables of weight-loss success in obese dieters. While many refer to gender, initial weight, or previous dieting as predictors, causality has not been established in these cases (Kiernan et al., 1998; Traverso et al., 2000; Teixeira et al., 2002, 2005). Of the various psychological constructs that have been studied, only self-motivation, or general efficacy, has yielded consistent findings (Williams et al., 1996; Teixeira et al., 2002, 2005; Palmeira et al., 2007). The explanation for this is that setting goals to oneself, and self-assurance in the ability to see the goals through, contribute to effective goal-directed behavior (Teixeira et al., 2002, 2005). These constructs seem to have some similarity to future-oriented thinking, yet they do not assess the processing of future consequences *per se*.

A potential limitation of the study is the fact that participants self-selected to participate in it. Yet, the sample was similar to the weight-management program's completer population in terms of gender, age, race/ethnicity, and weight-loss outcomes (see Table 1). Hence self-selection does not seem to be a major concern. It may be argued, though, that homogeneity in our subject pool is the reason why gender and initial weight did not predict weight loss in our study. This is in contrast with previous studies (e.g., Kiernan et al., 1998; Traverso et al., 2000; Diabetes Prevention Program Research Group, 2004), though other researchers have reported similar null results as well (Kiernan et al., 1998; Hollis et al., 2008). The fact that we did not control for eating disorders such as bulimia and binge eating is also a potential limitation, although bulimia nervosa has been found to be unrelated to recency (Chan et al., 2014). Finally, the analytical sample of 44 participants is quite modest in size. Therefore—and despite the significance of the results—the present findings should be regarded as preliminary and further replication is warranted.

An important issue that couldn't be addressed in the present study is gender differences. Men are less likely than women to seek treatment for obesity, and hence, to be included in obesity studies (Gray et al., 2009). Studies of decision-making in obese individuals also tend to be female-dominated (e.g., Nederkoorn et al., 2006; Batterink et al., 2010), as was the case in the present study. Yet, the few studies that compared between obese men and women concluded that they differ in their decision-making patterns, with obese men being less likely than obese women to display poor inhibitory control (Weller et al., 2008; Koritzky et al., 2012). Therefore, it is possible that difficulty to incorporate long-term considerations into decision-making is a more prevalent problem among obese women than among obese men. It follows that in order to obtain a complete picture of decision-making and interventions in obesity, men, and women should be studied separately.

Future, research should also address the links between recency and long-term weight-loss maintenance. It has been shown that maintaining a weight loss is remarkably challenging for many (e.g., Franz et al., 2007; Look AHEAD Research Group, 2014), and the role of cognitive factors in this process has not been completely understood (e.g., Williams et al., 1996; Hollis et al., 2008).

In sum, the present study shows that a cognitive/decision-making property—recency—predicts success in behavioral obesity treatments, and suggests a way by which doctors and healthcare professionals can identify the patients who are more likely to benefit from this treatment type. A major challenge faced by medical professionals is to select the right treatment for every patient. Because non-behavioral treatments for obesity—such as bariatric surgery—are costly, most patients are required by health insurance providers to try behavioral treatment at first. Because many patients do not benefit from behavioral treatment, this causes delays for them before they can achieve weight loss, which means a longer time will pass before their health can be improved. If physicians were able to predict a-priori who is likely (or unlikely) to benefit from behavioral treatment, they would be better able to match each patient with the treatment that is optimal for him or her. From this perspective, our findings underline the importance of moving beyond a “one size fits all” approach to weight-management research and practice. Not only do they add to professionals' ability to match patients to treatments effectively, but they also outline a way to facilitate weight-management processes for more patients. This can be done, for example, by encouraging clients to focus more on the long-term consequences of their choices. Although, theory posits that obesity is sustained by failure to incorporate long-term considerations into decision-making (Bechara, 2005; Carr et al., 2011), few attempts have been made to translate these notions into interventions (e.g., Higgs, 2002).

### Conflict of interest statement

The authors declare that the research was conducted in the absence of any commercial or financial relationships that could be construed as a potential conflict of interest.

## Footnotes

^1^The criterion for obesity in terms of BMI is < 30. The majority of those who lost weight successfully (9 out of 12) have remained with BMIs of over 30 after the intervention. Two participants have shifted into the 25 ≤ BMI < 30 “overweight” category, and one person shifted into the BMI < 25 “normal weight” category. None of the unsuccessful participants' BMI category has shifted.
